# Is there a connection between Mental Health issues and poverty? A service evaluation in East Norfolk and Suffolk, UK

**DOI:** 10.1192/j.eurpsy.2025.1118

**Published:** 2025-08-26

**Authors:** D. Collins, J. Beezhold

**Affiliations:** 1 CFYP, NHS, Norwich; 2 CYFP, NSFT, Great Yarmouth, United Kingdom

## Abstract

**Introduction:**

It is well established that living, or growing up, in poverty has a negative impact on both physical and mental health. The area our service covers includes Great Yarmouth and Lowestoft, 2 of the most economically impoverished areas of the UK. The vast majority of our patient group will have grown up in relative poverty. While there are associations between poverty and impaired physical health and increased risk of some mental health conditions, the actual causal link is unclear.

**Objectives:**

To explore if there appears to be a link between growing up in poverty and developing a significant mental illness.

**Methods:**

Data anlaysis from Consultant caseload list.

We do know that there are some factors associated with both poverty and increased risk of mental illness and these include;

- Parental drug or alcohol abuse
- Parental mental health problems (if these are not well managed)
- Early/premature death of a parent
- Exposure to domestic violence
- Physical abuse
- Going into the Care System
- Early drug or alcohol use
- Early separation or loss of a parent

**Results:**

**Findings**

Total number of patients = 122

Number who have a specific factor associated with poverty =56

This equates to 46% of my current caseload.

**Gender** =35 female (62.5), male 21 (37.5%)

**Conclusions:**

**Summary of Findings** – “*The poor bear the greatest burden of mental illness” (2)*

This would certainly seem to be the case, from the findings of this service evaluation. Our findings show that a significant percentage of our patient group have mental health issues directly related to poverty.

It is worth noting that the vast majority of my patient case load grew up in poverty, due the demographics of the area we work in (a quick analysis suggests about 97% of my case load are from working class, impoverished backgrounds). We abandoned recording “parental unemployment” in this analysis, because for all but a few, this was the case. Unemployment is an entrenched issue in this area, with the demise of the shipping and offshore industries, currently standing at 5.4% in Yarmouth and 3.5% in Lowestoft (3) (National average 3.8%). For those that are employed, poverty is a significant issue with many in low paid jobs. I have also not included here factors associated with poverty, such as poor diet, smoking, malnutrition, poor dentition, and obesity, but we know these are the case for many patients seen here.

**Disclosure of Interest:**

None Declared

